# Effect of ticagrelor versus clopidogrel after implantation of drug-eluting stents guided by either intravascular ultrasound or angiography in patients with acute coronary syndrome—propensity score matching analysis

**DOI:** 10.1186/s12872-023-03659-0

**Published:** 2024-01-18

**Authors:** Yinan Zhao, Yuxin Yang, Lei Guo, Dapeng Shen, Zhichao Dong, Yajuan Lin, Hao Liu, Yushan Wei, Bo Zhang

**Affiliations:** 1 Department of Cardiology, First Affiliated Hospital of Dalian Medical University, Dalian, 116011 China; 2 Department of Cardiology, Fuxin center Hospital, Fuxin, 123099 China; 3 Department of Scientific research, The First Affiliated Hospital of Dalian Medical University, Dalian, 116011 Liaoning China

**Keywords:** Angiography, Clopidogrel, Dual antiplatelet, Intravascular ultrasound, Ticagrelor

## Abstract

**Background:**

The effect of different dual antiplatelet therapies on thrombotic events on the background of intravascular ultrasound (IVUS) guidance is unclear. We investigated whether ticagrelor can provide any additional benefit to clopidogrel in reducing thrombotic events in acute coronary syndrome (ACS) treated with drug- eluting stent (DES), when guided by IVUS or not.

**Methods:**

A total of 5,666 ACS patients who underwent DES implantation and who were discharged on dual antiplatelet therapy were enrolled and grouped according to the use of IVUS or not. Each group was subdivided into two subgroups according to the type of P2Y12 inhibitor used after discharge. Propensity score matching (PSM) was used between the IVUS and no-IVUS groups. Covariate adjustment of Cox proportional hazards model was used between the ticagrelor and clopidogrel groups. Thrombotic event at 12 months was compared in groups separately.

**Results:**

After PSM, 12-month follow-up data were available for 1,174 patients. Major adverse cardiac events (MACE) were less frequent in the IVUS-guided group (2.2% vs. 4.3%, *P* = 0.081) with a trend toward statistical significance. Comparison of antiplatelet regimens revealed significantly fewer major adverse cardiac and cerebrovascular events (MACCE) with ticagrelor in the entire PSM cohort and angiography-guided subgroup (2.9% vs. 5.7%, *P* = 0.035; 3.1% vs. 6.4%, *P* = 0.020, respectively). Among patients in the IVUS-guided group the outcome was comparable (2.5% vs. 4.4%, *P* = 0.312). Ticagrelor was associated with increasing bleeding incidence in the entire PSM cohort (1.3% vs. 3.3%, *P* = 0.030), mainly due to Bleeding Academic Research Consortium type 2 bleeding (0.7% vs. 2.6%, *P* = 0.010). The results were consistent after covariate adjustment of Cox proportional hazards model.

**Conclusion:**

The comparison of ischemic benefit between ticagrelor and clopidogrel was similar in patients receiving IVUS guidance during stent implantation, probably due to the precise implantation of IVUS. Multicenter, randomized studies should be performed to validate this conclusion.

**Supplementary Information:**

The online version contains supplementary material available at 10.1186/s12872-023-03659-0.

## Background

Dual antiplatelet therapy (DAPT) with aspirin and a P2Y12 inhibitor is the cornerstone anti-thrombosis treatment for patients with acute coronary syndrome (ACS) who undergo stent implantation.Although the more potent P2Y12 inhibitor, ticagrelor, can significantly reduce the ischemic events in ACS patients compared to clopidogrel [1], the high bleeding risk and other side effects associated with ticagrelor have limited its clinical application [2, 3]. The benefit between the two agents has remained controversial in real-world studies [4–6]. Thus, clopidogrel is still extensively prescribed.

Intravascular ultrasound (IVUS) also reduces thrombosis events after stent implantation [7–10],while further analyses of the effects of different antiplatelet therapies on the outcomes in the studies has not been done. Therefore, the differences between the results of the comparison between clopidogrel and ticagrelor in these two different conditions with or without IVUS is not yet clear. We were curious about if IVUS-guided stents implantation could reduce the need for intensive post-operative antithrombotic therapy,which might address treatment options in patients with high blood risk or intolerance to ticagrelor.

The purpose of this study was to investigate whether ticagrelor could provide any additional benefit to clopidogrel in terms of clinical outcomes in ACS treated with drug-eluting stents (DES) under IVUS or angiographic guidance.

## Methods

### Population study design

This population-based retrospective observational cohort study was conducted between 01 January 2016 to 01 January 2021 at the cardiovascular department of the First Affiliated Hospital of Dalian Medical University (FAHDM), Dalian City, Liaoning Province of China. The clinical data for this study were retrieved from the Electronic Medical Record Research Database (EMRRD) (YiDu Cloud technology Ltd, Beijing, China) of FAHDM. The EMRRD is established to create a computerized clinical archive,where clinical notes are regularly updated.

We recruited 5,666 patients who had been diagnosed with ACS implanted with one of more DES guided by angiography or IVUS, and all patients received ticagrelor/clopidogrel and aspirin after discharge. Exclusion criteria included previous coronary artery bypass graft, long-term use of anticoagulants, hepatic dysfunction, malignant tumor, and death before discharge. If any variables related to baseline information, lesion, procedure were incomplete for a patient, they were considered as data missing and the patient was excluded.

### Procedure characteristic and dual antiplatelet therapy

**Fig. 1:**
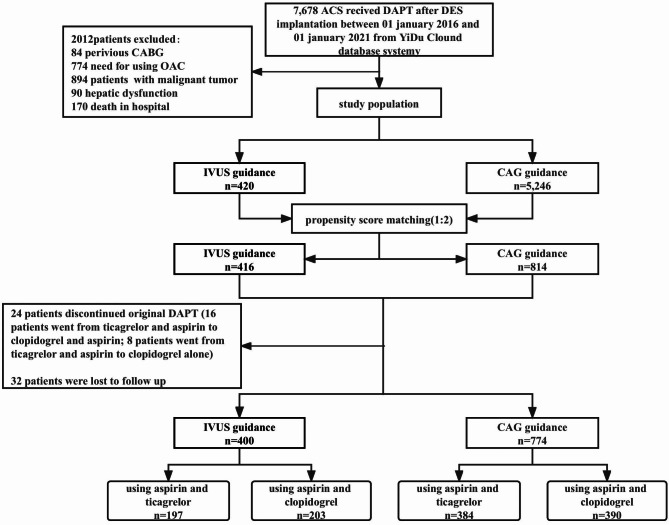
Flowchart of the study Abbreviation: ACS, acute coronary syndrome; CABG, coronary artery bypass graft; CAG, coronary angiography; DAPT, dual antiplatelet therapy; DES, drug eluting stent; IVUS, intravascular ultrasound; OAC, oral anticoagulant

In the angiography-guided group, each stent was placed only with angiography guidance. In the IVUS-guided group, stent placement included angiography and IVUS guidance. IVUS could be performed before, during or after percutaneous coronary intervention (PCI). Everyone was discharged on clopidogrel 75 mg once daily or ticagrelor 90 mg twice daily with aspirin 100 mg once daily, regardless of the antiplatelet regimen used during hospitalization. The utility of IVUS and the choice of antiplatelet agents were at the discretion of the operator. All patients were divided into IVUS-guided group and angiography-guided group. Each group was subdivided into two subgroups according to the type of P2Y12 inhibitor used after discharge. The flowchart is provided in Fig. 1.

### Endpoints and definitions

All deaths were regarded as cardiovascular death unless there was an unequivocal non-cardiac cause could be established. We defined Myocardial infarction (MI) according to the consencus defination of the socieaty cardiovascular angiography and inventation [11]. Ischemic stroke was defined as newly developed neurologic symptoms and required hospitalization.

The primary objective was to explore whether ticagrelor can provide any additional benefit to clopidogrel in reducing thrombotic events in ACS treated with DES, when guided by IVUS or not. For this purpose, the endpoint for ticagrelor vs. clopidogrel was defined as major adverse cardiac cerebrovascular events (MACCE), including cardiac death, MI and ischemic stroke.

The secondary objective was to access the effect of IVUS on reducing thrombotic events compared with angiography. For this purpose, the endpoint for IVUS vs. angiography was defined as major adverse cardiac events (MACE), including MI and cardiac death.

The safety endpoint was defined as Bleeding Academic Research Consortium (BARC) type 2, 3, and 5 bleeding [12]. Clinical follow-up was done with telephone interviews .

We uniformly conducted follow-up from February 1 to February 20, 2022. The follow-up time of some patients exceeded the follow-up time window, due to the large time span of enrolled patients.

### Statistical analysis

All continuous variables are expressed as mean ± standard deviation or median (Q1-Q3), and compared with the t test or Mann-Whitney U test as appropriate. Categorical variables are described as counts (percentage) and analyzed with Fisher’s exact test or chi-squared test.

Propensity score matching (PSM) was used to balance the baseline, lesion, and procedural features between the IVUS-guided and angiography-guided groups (including age, sex, hypertension, diabetes, smoker, dyslipidemia, prior stroke, chronic kidney disease, prior myocardial infarction, prior percutaneous coronary intervention, diagnose of acute coronary syndrome, multivessel, chronic total occlusion, left main coronary, number of stents, stent length, stent diameter, left ventricular ejection fraction, the level of low density lipoprotein, the level of hemoglobin). A 1:2 nearest neighbor matching was employed and the caliper of width was equal to 0.2. Any comparison was analyzed in the propensity score-matched cohort.

The incidence of endpoint for IVUS vs. angiography and ticagrelor vs. clopidogrel at 12 months follow-up was estimated by the Kaplan-Meier method. Differences in survival were compared using the log-rank test. The hazard risk and 95% confidence interval were obtained with Cox proportional hazards models. Covariate adjustment of multivariate Cox was used to adjust the baseline, lesion, and procedural features between the ticagrelor and clopidogrel groups.

Results were considered significant at *P* < 0.05, PSM was performed using R. software. Additional analyses used SPSS Statistics v25.0 (SPSS Inc., Chicago, IL, USA).

## Results

### Baseline characteristic

A total of 5,666 patients were eligible for enrollment. PSM between the IVUS and angiography-guided groups involved 1,230 patients with well-balanced clinical characteristics (Table 1). Of the 1,230 patients, 34 patients were lost to follow-up and 23 patients discontinued the original DAPT regimen and were absent from the final analysis. Of the 23 patients, 12 had shortness of breath, 5 had a dry cough, and 6 had switched to other P2Y12 inhibitors for financial reasons. Patients who changed medication due to severe bleeding were considered to be the end point and follow-up was discontinued.Thus, the 12-month follow-up included 1174 patients (400 patients in the IVUS guidance group, 774 patients in the angiography guidance group). All variables between the two groups were still balanced (Additional file 1). There was a higher prevalence of left main coronary disease and lower prevalence of prior ischemic stroke in the IVUS-guided group with ticagrelor. Furthermore, the hemoglobin value was higher and more and longer stents were implanted (Table 2). In the angiography-guided group, patients receiving ticagrelor were younger, predominantly women, with lower proportions of prior ischemic stroke and unstable angina pectoris, higher proportions of smokers, non-ST elevation myocardial infarction, chronic total occlusion, left main disease, and the hemoglobin values (Table 2).

**Table 1:** Baseline characteristics between IVUS- and angiography-guided groups

	Before matching			After matching		
	IVUS-guided N = 420	Angiography-guided N = 4246	*p*-value	IVUS-guided N = 416	Angiography-guided N = 814	*p*-value
Age	67(61–74)	67(59–74)	0.531	67(61–74)	67(61–74)	0.745
Female	77(18.3)	320(25.2)	0.002	77(18.5)	158(19.4)	0.704
Hypertension	265(63.1)	3008(57.3)	0.022	261(62.7)	514(63.1)	0.889
Diabetes	139(33.1)	1527(29.1)	0.084	137(32.9)	268(32.9)	0.998
Smoker	202(48.1)	2370(45.2)	0.248	200(48.1)	364)44.7)	0.862
Dyslipidemia	24(5.7)	345(6.6)	0.491	24(5.8)	43(5.3)	0.722
Prior stroke	31(7.4)	130(2.5)	<0.001	29(7.0)	54(6.6)	0.823
CKD	10(2.4)	105(2.0)	0.596	10(2.4)	25(3.1)	0.505
Prior MI	60(14.3)	647(12.3)	0.244	60(14.4)	110(13.5)	0.662
Prior PCI	72(17.1)	490(9.3)	<0.001	68(16.3)	126(15.5)	0.693
Diagnosis of ACS						
STEMI	53(12.6)	1386(26.4)	<0.001	53(12.7)	103(12.7)	0.965
NSTEMI	101(24.0)	1223(23.3)	0.732	101(24.3)	183(22.5)	0.479
UA	266(63.3)	2637(50.3)	<0.001	262(63)	528(64.9)	0.514
Multivessel	318(75.7)	3999(76.2)	0.811	314(75.5)	633(77.8)	0.368
CTO	40(9.5)	442(8.4)	0.438	40(9.6)	87(10.7)	0.559
LMCA	84(20.0)	297(5.7)	<0.001	80(19.2)	144(17.7)	0.508
LVEF(%)	58 (55–59)	58(53–59)	0.026	58(55–59)	58(55–59)	0.901
LDL-C(mmol/L)	2.4 (1.8-3.0)	2.7(2.1–3.5)	<0.001	2.4(1.8-3.0)	2.4(1.9-3.0)	0.378
WBC(10^9)	6.8(5.7–8.3)	7.4(6.1–9.3)	<0.001	6.8(5.7–8.3)	7.0(5.9–8.6)	0.146
Hb(g/L)	14.1(13–15)	14.1(13–15)	0.510	14.1(13–15)	14.1(13–15)	0.305
PLT(10^9/L)	205(174–280)	209(178–246)	0.315	205(175–246)	206(174–243)	0.779
Crea(umol/L)	72(62–83)	71(61–82)	0.374	71(62–83)	73(63–85)	0.110
Ticagrelor	209(49.8)	2822(53.9)	0.111	207(49.8)	408(50.1)	0.904
NO. of stent	1.5 ± 0.8	1.6 ± 0.8	0.055	1.5 ± 0.8	1.6 ± 0.8	0.078
Total stent length(mm)	39.2 ± 23.8	41.8 ± 24.3	0.033	39.3 ± 23.8	41.6 ± 23.7	0.114
Mean stent diameter(mm)	3.2 ± 1.1	3.1 ± 0.7	< 0.001	3.2 ± 1.1	3.1 ± 0.5	0.001

*ACS*, acute coronary syndrome; *CAG*, angiography; *CKD*, chronic kidney disease; *CTO*, chronic total occlusion; *Hb*, hemoglobin; *LMCA*, left main coronary; *LVEF*, left ventricular eject fraction; *LDL-C*, low density lipoprotein cholesterol; *MI*, myocardial infarction; *NSTEMI*, non-ST-segment elevation myocardial infarction; *PCI*, percutaneous coronary intervention; *PLT*, platelet; *UA*, unstable angina pectoris; *WBC*, white blood cell; *STEMI*, ST-segment elevation myocardial infarction

**Table 2:** Baseline characteristics grouped according to type of P2Y12 inhibitor after propensity score matching

	IVUS guidance group			CAG guidance group		
	Ticagrelor N = 197	Clopidogrel N = 203	*p*-value	Ticagrelor N = 384	Clopidogrel N = 390	*p*-value
Age	60 (60–72)	69 (63–75)	0.005	66 (59–71)	69 (63–77)	<0.001
Female	33(16.8)	40(19.7)	0.445	47(12.2)	101(25.9)	<0.001
Hypertension	115(58.4)	137 (67.5)	0.059	228(59.4)	259(66.4)	0.043
Diabetes	62(31.5)	71(35)	0.457	122(31.8)	134(34.4)	0.444
Smoker	98(49.7)	94(46.3)	0.491	197(51.3)	154(39.5)	0.001
Dyslipidemia	12(6.1)	11(5.4)	0.773	25(6.5))	15(3.8)	0.094
Prior stroke	3(1.5)	25(12.3)	<0.001	16(4.2)	35(9.0)	0.007
CKD	4(2.0)	6(3.0)	0.553	10(2.6)	12(3.1)	0.692
Prior MI	30(15.2))	29(14.3)	0.790	52(13.5)	54(13.8)	0.902
Prior PCI	38(19.3)	27(13.3)	0.105	64(16.7)	55(14.1)	0.323
Diagnosis of ACS						
STEMI	19(9.6)	32(15.8)	0.067	48(12.5)	46(11.8)	0.764
NSTEMI	50(25.4)	47(23.2)	0.603	105(27.3)	72(18.5)	0.003
UA	128(65.0)	124(61.1)	0.420	231(60.2)	272(69.7)	0.005
Multivessel	156(79.2)	145(71.4)	0.072	293(76.3)	308(79.0)	0.372
CTO	19(9.6)	18(8.9)	0.788	51(13.3)	32(8.2)	0.022
LMCA	49(24.9)	26(12.8)	0.002	87(22.7)	52(13.3)	0.001
LVEF(%)	58 (54–59)	58 (55–59)	0.346	58 (55–59)	58 (55–59)	0.164
LDL-C(mmol/L)	2.4 (1.8-3.0)	2.4 (1.9–3.1)	0.707	2.4 (1.8-3.0)	2.45(2.0–3.0)	0.083
WBC(10^9)	7.0(5.7–8.7)	6.8(5.7–8.1)	0.410	7.0(5.9–8.5)	7.0(5.8–8.6)	0.705
Hb(g/L)	14.2 (13–15)	13.8(13–15)	0.023	14.3 (13–15)	13.8(13–15)	<0.001
PLT(10^9/L)	205 (174–245)	204 (177–248)	0.857	207 (176–243)	205 (172–244)	0.466
Crea(umol/L)	72(62–83)	72(62–83)	0.590	73(63–84)	72(61–85)	0.357
NO. of stent	1.6 ± 0.9	1.4 ± 0.7	0.042	1.6 ± 0.9	1.6 ± 0.8	0.804
Total stent length(mm)	41.9 ± 27.4	37.1 ± 19.9	0.044	42.1 ± 24.2	40.6 ± 22.7	0.375
Mean stent diameter(mm)	3.2 ± 0.5	3.3 ± 1.5	0.496	3.1 ± 0.5	3.0 ± 0.5	0.060

*ACS*, acute coronary syndrome; *CAG*, angiography; *CKD*, chronic kidney disease; *CTO*, chronic total occlusion; *Hb*, hemoglobin; *LMCA*, left main coronary; *LDL-C*, low density lipoprotein cholesterol; *MI*, myocardial infarction; *NSTEMI*, non-ST-segment elevation myocardial infarction; *PCI*, percutaneous coronary intervention; *PLT*, platelet; *UA*, unstable angina pectoris; *WBC*, white blood cell; *STEMI*, ST-segment elevation myocardial infarction

### Clinical endpoint

**Fig. 2:**
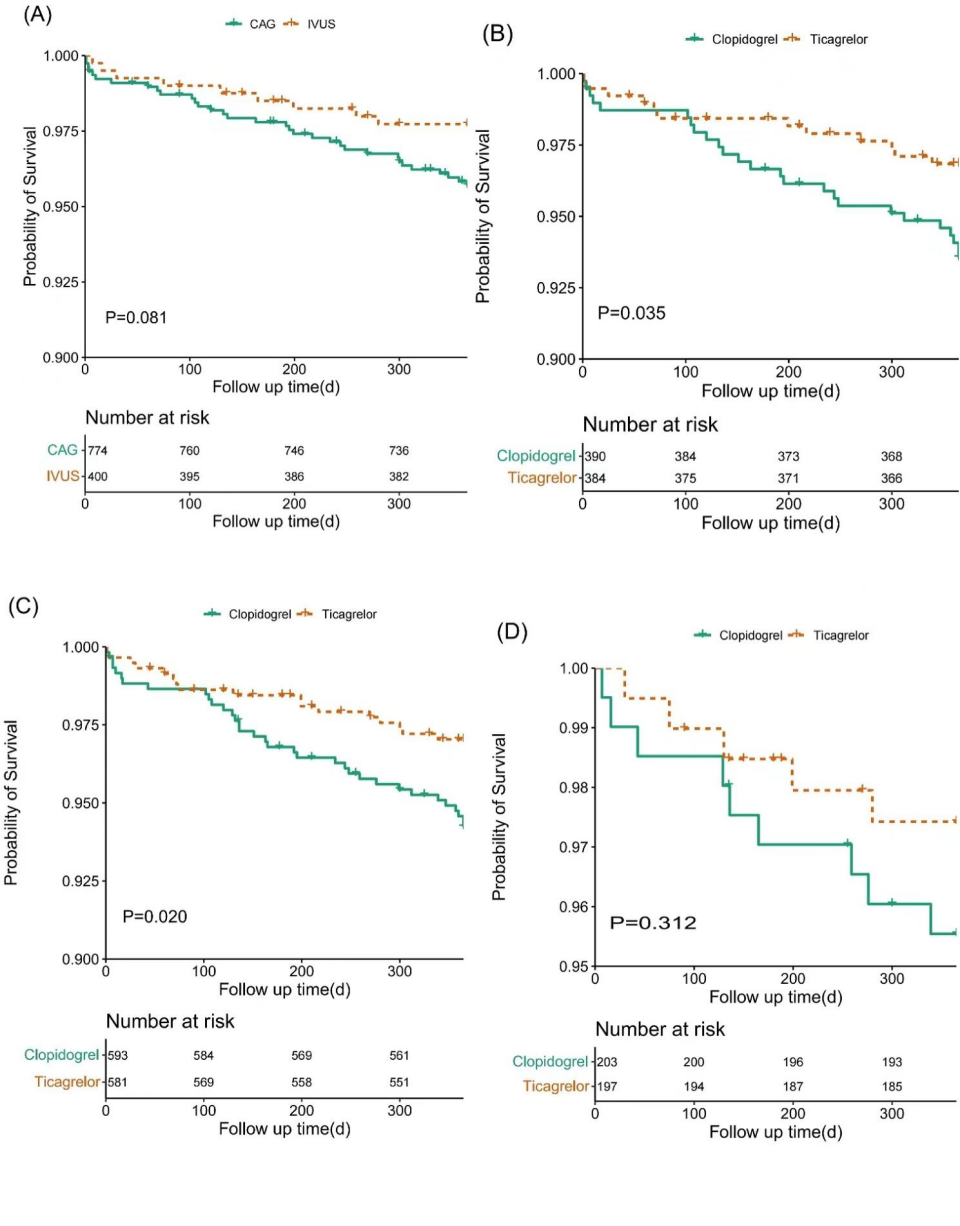
Kaplan-Meier survival curve of primary endpoint at follow-up after propensity score matching. (**A**) Compared to the angiography guidance group, there was a tendency to reduce the rate of MACE in the IVUS guidance group. (**B**-**D**) Compared to the clopidogrel group, MACCE was lower in the ticagrelor group both in the whole cohort (**B**) and the angiography-guided subgroup (**C**), while in the IVUS-guided subgroup MACCE was similar between the two antiplatelet treatments (**D**) Abbreviation: CAG, angiography; IVUS, intravascular ultrasound; MACCE, major adverse cardiac and cerebrovascular event; MACE, major adverse cardiac event

**Fig. 3:**
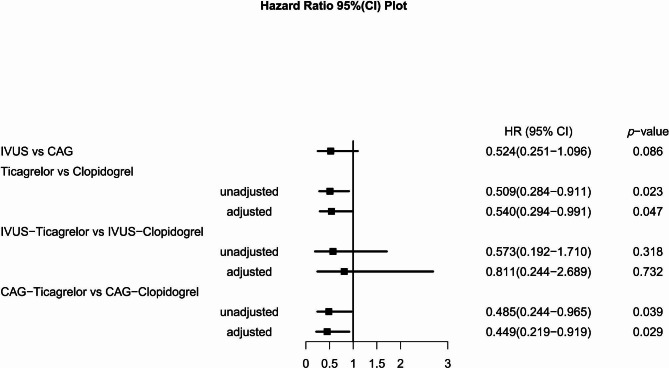
Hazard ratio (HR) for primary endpoint in groups before and after multivariate Cox proportional hazards models correction Abbreviation: CAG, angiography; CI, confidence interval; IVUS, intravascular ultrasound

After PSM, IVUS guidance was associated with reduced rate of MACE (2.2% vs. 4.3%, *P* = 0.081) and MI (1% vs. 2.7%, *P* = 0.055) with a trend toward statistical significance at 12 months. Considering the comparison between ticagrelor and clopidogrel, the rate of the MACCE was significantly lower with ticagrelor (2.9% vs. 5.7%, *P* = 0.020) at 12 months. When comparing the outcome in subgroups where IVUS was used or not used, the difference was also significant in the angiography-guided subgroup (3.1% vs. 6.4%, *P* = 0.035). Rather, it was similar in the IVUS-guided subgroup (2.5% vs. 4.4%, *P* = 0.312). These results were consistent after covariate adjustment. No significant difference was observed for individual endpoints in groups. Kaplan-Meier survival curves are shown in Fig. 2. The endpoint events at 12 months between ticagrelor and clopidogrel after covariate adjustment of Cox proportional hazards model are shown in Table 3. Hazard risks for the primary endpoint in groups before and after adjustment are shown in Fig. 3.

**Table 3:** Endpoint events at 12 months after covariate adjustment of Cox proportional hazards models

Entire study population events	Ticagrelor (N = 581)	Clopidogrel (N = 593)	HR (95% CI)	p*-*value
MACCE	17 (2.9%)	34 (5.7%)	0.540 (0.294–0.991)	0.047
Cardiac death	5 (0.9%)	12 (2%)	0.422 (0.143–1.247)	0.118
MI	11(1.9%)	14 (2.4%)	0.814 (0.356–1.863)	0.627
Ischemic stroke	1 (0.2%)	8 (1.3%)	0.175 (0.021–1.462)	0.107
Bleeding(BARC2,3,5)	19(3.3%)	8(1.3%)	3.322 (1.397–7.898)	0.007
BARC 2	15 (2.6%)	4 (0.7%)	-	0.011
BARC 3	2 (0.3%)	4 (0.7%)	-	0.907
BARC 5	2 (0.3%)	0	-	0.967
**CAG-guided subgroup events**	**Ticagrelor (N = 384)**	**Clopidogrel (N = 390)**	**HR(95% CI)**	***p***-**value**
MACCE	12 (3.1%)	25 (6.4%)	0.449 (0.219–0.919)	0.029
Cardiac death	3 (0.8%)	9 (2.3%)	0.267 (0.069–1.028)	0.055
MI	9 (2.3%)	12 (3.1%)	0.778 (0.314–1.928)	0.588
Ishemic stroke	0	4(1%)	-	0.956
**IVUS-guided subgroup events**	**Ticagrelor (N = 197)**	**Clopidogrel (N = 203)**	**HR(95% CI)**	***p***-**value**
MACCE	5 (2.5%)	9 (4.4%)	0.811 (0.244–2.689)	0.732
Cardiac death	2 (1%)	3 (1.5%)	0.801 (0.082–7.850)	0.849
MI	2 (1%)	2 (1%)	1.028 (0.123–8.567)	0.980
Ischemic stroke	1 (0.5%)	4 (2%)	0.536 (0.050–5.804)	0.608

*BARC*, Bleeding Academic Research Consortium; *CI*, confidence interval;*CAG*, angiography; *HR*, hazard ratio; *IVUS*, intravascular ultrasound; *MACE*, major adverse cardiac event; *MACCE*, major adverse cardiac and cerebrovascular event; *MI*, myocardial infarction

### Safety Endpoint

Compared with clopidogrel, the incidence of primary safety endpoints (BARC2, BARC3, and BARC5) was higher when patients were treated with ticagrelor (1.3% vs. 3.3%, *P* = 0.003), mainly due to BARC2 bleeding (0.7% vs. 2.6%, *P* = 0.001), there were no variances in BARC3 and BARC5 (0.7% vs. 0.7%, *P* = 0.995) between the two groups.

## Discussion

There are three main outcomes in the present study. In the PSM cohort, (1) IVUS was associated with decreased MACE, but without statistical significance. (2) Ticagrelor significantly lowered the risk of MACCE compared with clopidogrel, especially in the angiography-guided group. The result differed slightly in the IVUS-guided group. (3) Ticagrelor increased the overall bleeding risk, mainly driven by BARC2 bleeding.

Recurrent thrombotic events after PCI tend to be associated with MI and death [13]. Ticagrelor has been recommended after PCI, given its more potent antiplatelet effect compared with clopidogrel [14, 15]. However, ticagrelor often poses a higher risk of bleeding [2, 16], including in our study. In fact, we also observed discrepancies regarding the advantage of ticagrelor in reducing thrombotic events over clopidogrel in the subgroup of patients with different bleeding and ischemia risks. [4, 17].

Patients treated with IVUS could be seen as at low risk for ischemia. Mechanical factors, such as under-expansion, malapposition, edge dissection, or residual plaque burden, may be correlated with stent thrombosis [18, 19]. IVUS could detect and fix these mechanical complications to reduce thrombotic events. Also, in the present study, IVUS-guided DES implantation tended to reduce MACE compared to angiography guidance, although the difference did not reach statistical significance. One explanation for this result could be that the MLA was not analyzed, which is a crucial predictor of stent thrombosis [20], since this data was incomplete in our operative recordings. Another explanation could be unplanned revascularization was not included in our MACE, this may underestimate the effect of IVUS. Finally, in most previous IVUS studies, DAPT with clopidogrel and aspirin were prescribed after PCI [7, 21]. Even when more potent P2Y12 inhibitor was used, the proportion was low. A recent study that compared the hard endpoint of cardiac death and MI between IVUS and angiography at 3 years, ticagrelor and prasugrel was used in the study, while the total proportion was less than 40% [9]. Meanwhile, although the benefit of IVUS was clarified in these studies, further analysis regarding the influence of different antiplatelet regimens on the clinical outcomes was not performed. As antiplatelet agents that can also reduce thrombotic events, different DAPT might affect the outcomes of studies on IVUS. Notably, ticagrelor accounted for nearly half both in the IVUS-guided group and the angiography-guided group in our PSM cohort. The superiority of ticagrelor over clopidogrel was also confirmed in our study, therefore, the improvement in thrombotic events with ticagrelor might be associated with the absence of a statistically significant difference of MACE in the IVUS-guided group.

The present findings in our study also indicate that ticagrelor could not be always recommended to patients who receive implanted stents with IVUS guidance and who are at low risk for ischemia, given the higher risk of bleeding with the use of the more potent antiplatelet agent. When patients who did or did not use IVUS was analyzed separately in our study, the efficacy of ticagrelor was more obvious in the angiography-guided subgroup, clopidogrel seems also to be a safe agent after stent implantation with IVUS, not only ticagrelor. This result supplies clinicians with new idea for an antiplatelet regimen alternative when balancing the treatment regimen with the risks of bleeding and thromboembolic events after PCI-DES. Indeed, concerns about the increased bleeding risk in special patients often lead clinicants to prescribe a less potent P2Y12 inhibitor, potentially leaving patients undertreated and exposing them to a higher incidence of thrombotic complications. Therefore, to better balance the ischemia and bleeding risk, our study result might provide the new ideas with clinicians.

Different from that in the CAG group, there were several explanations for the closer difference between ticagelor and clopidogrel in the IVUS group. First, patients who treated with DES implantation with IVUS guidance were considered at low risk of ischemia due to decreased thrombogenic factors. IVUS could lead a larger MLA, which might in turn improve blood flow and microcirculation. In one study, the favorable effect of ticagrelor on MACE was deemed to be partly owing to the inhibition of adenosine and the consequent improved microcirculation [22]. Therefore,the microvessel benefit of IVUS weakened the microvessel benefit of ticagrelor in patients who receive stents with IVUS guidance.

In addition, when endothelial cells are injured, subcutaneous components were released in the bloodstream and some changes of the mechanical microenvironment caused by stents can contribute to platelet aggregation [23]. Therefore, a more potent antiplatelet agent was recommended for preventing thrombosis after PCI [15]. IVUS could precisely guide stents and balloons to the target lesion. Although IVUS tends to be used with more complex procedures involving longer and larger stents with larger balloons and higher inflation pressure, no increased thrombotic complications have been described [24]. The precise PCI strategy achieved by IVUS with mild damage to the vascular wall might reduce injury and inflammation of endothelial cells. Thus, IVUS guidance can reduce platelet aggregation and thrombogenesis, which could further alleviate the demand for potent platelet agents. This deduction was supported by the findings of the ADAPT study [25], which demonstrated a significantly low risk of thrombotic events in patients whose stents were guided by IVUS versus those guided by angiography. The less potent P2Y12 inhibitor, clopidogrel, was prescribed for the whole study cohort and clopidogrel hypo-responsiveness was more frequently seen in the IVUS-guided group [25]. Our comparative data obtained by matching patients concerning stent size still revealed a beneficial tendency with IVUS. To some extent, IVUS attenuated the need for strong antithrombotic therapy.

Finally, except for the target lesion, recurrent thrombotic events arise from high-risk plaques at non-culprit lesions, which are characterized by a large plaque burden, small MLD, and thin-cap fibroatheromas [26]. IVUS can detect these high-risk plaques outside stents, which can drive the selection of a more appropriate medical management to decrease residue risk [27]. This personalized medicine management in the IVUS group might weaken the difference between ticagrelor and clopidogrel.

Recent evidence suggested that after an initial period of DAPT, P2Y12 inhibitor monotherapy can be preferable to aspirin monotherapy for long-term secondary prevention in patients undergoing PCI, regardless of ticagrelor or clopidogrel [28]. In our study, there is little difference between ticagrelor and clopidogrel for patients with IVUS. Not hard to find, study on antithrombotic therapy is ongoing. In clinical practice, achieving a good balance between the risk of ischemia and bleeding in patients treated with dual antiplatelet therapy is not an easy task. Physicians now have to deal with this every day. Ticagrelor was recommended for patients after stent implantation, however, the high bleeding risk associated with ticagrelor limited its application. Intravascular ultrasound (IVUS) has been shown to reduce thrombotic events. However,the clinical outcomes of ticagrelor over clopidogrel based on IVUS-guided PCI for patients with ACS has been unclear. Our findings provide insight on the choice between ticagrelor and clopidogrel for clinicians. Clopidogrel may also be a safe and suitable alternative for patients with ACS whose stent implantation is guided by IVUS. This study result may provide further ideas for personalized treatment.

### Limitations

This was a single central observational study. Although the baseline, lesion, and procedural features were well balanced using PSM, unknown confounders were possible. First, the decision whether to use IVUS guidance and IVUS guidance criteria were solely at the operators’ discretion. Second, the endpoint of MI could not be conclusively determined in the same treated vessel guided with IVUS. Third, our study was conclusive only for the composite endpoint related to thrombotic events. Difference for individual endpoints did not reach statistical significance. Fourth, as a retrospective study, there might be recall bias in the outcome of the endpoint event. At last, the current study result would only be hypothesis-generating.

## Conclusions

In ACS patients implanted with a DES, the comparison of ischemic benefit between ticagrelor and clopidogrel was similar in patients receiving IVUS guidance during stent implantation. Multicenter, randomized studies should be performed to validate this conclusion.

### Electronic supplementary material

Below is the link to the electronic supplementary material.

Supplementary Material 1

## Data Availability

All datasets analyzed during the current study is available from the corresponding author on reasonable request.
