# Supplementary material for: Effect of ticagrelor versus clopidogrel after implantation of drug-eluting stents guided by either intravascular ultrasound or angiography in patients with acute coronary syndrome—propensity score matching analysis
Source: BMC Cardiovasc Disord. 2024 Jan 18;24:58. doi: 10.1186/s12872-023-03659-0 (PMC10795401; doi:10.1186/s12872-023-03659-0)
Supplement: Supplementary file 1 — Supplementary Material 1 [file 12872_2023_3659_MOESM1_ESM.docx]

**Title：Effect of ticagrelor versus clopidogrel after implantation of drug-eluting stents guided by either intravascular ultrasound or angiography in patients with acute coronary syndrome——Propensity score matching analysis**

**Authors :**Yinan Zhao^1,a^, Yuxin Yang^1,a^, Lei Guo^1^, Dapeng Shen^2^, Zhichao Dong^1^, Yajuan Lin^1^, Hao Liu^1^, Yushan Wei ^3,^*, Bo Zhang^1,^**

^1^ Department of Cardiology, First Affiliated Hospital of Dalian Medical University, Dalian, 116011, China

^2^ Department of Cardiology, Fuxin center Hospital, Fuxin, 123099, China

^3^ Department of Scientific research, The First Affiliated Hospital of Dalian Medical University, Dalian, Liaoning, 116011, China.

**** Corresponding author**

Bo Zhang, MD, PhD, Department of Cardiology, First Affiliated Hospital of Dalian Medical University, Dalian, 116011, China. E-mail: [dalianzhangbo@yahoo.com](mailto:dalianzhangbo@yahoo.com) (B. Zhang).

**Supplements**

Additional file 1. Characteristics for IVUS- and CAG-guided groups of patients included in the final analysis.

|  | IVUS-guided  （N=400） | Angiography-guided  （N=774） | P-value |
| --- | --- | --- | --- |
| Age | 67 (61-74) | 68 (60-74) | 0.785 |
| Male | 327(81.8) | 626(80.9) | 0.717 |
| Hypertension | 252(63.0) | 487(62.9) | 0.979 |
| Diabetes | 133(33.3) | 256(33.1) | 0.952 |
| Smoker | 192(48) | 351(45.3) | 0.388 |
| Dyslipidaemia | 23(5.8) | 40(5.2) | 0.675 |
| Prior stroke | 28(7.0) | 51(6.6) | 0.790 |
| CKD | 10(2.5) | 22(2.8) | 0.733 |
| Prior MI | 59(14.8) | 106(13.7) | 0.622 |
| Prior PCI | 65(16.3) | 119(15.4) | 0.696 |
| Diagnosis of ACS |  |  |  |
| STEMI | 51(12.8) | 94(12.1) | 0.765 |
| NSTEMI | 97(24.3) | 177(22.9) | 0.596 |
| UA | 252(63.0) | 503(65.0) | 0.501 |
| Multivessel | 301(75.3) | 601(77.6) | 0.356 |
| CTO | 37(9.3) | 83(10.7) | 0.430 |
| LCMA | 75(18.8) | 139(18) | 0.739 |
| Ticagrelor | 197(49.3) | 384(49.6) | 0.906 |
| EF% | 58 (55-59) | 58 (55-59) | 0.856 |
| LDL-C(mmol/L) | 2.4 (1.8-3.0) | 2.4 (1.9-3.0) | 0.453 |
| WBC(10^9) | 7.0(5.9-8.6) | 6.9(5.8-8.4) | 0.252 |
| Hb(g/L) | 141 (130-150) | 141 (131-151) | 0.395 |
| Crea(umol/L) | 72(62-83) | 72(62-84) | 0.201 |
| PLT(10^9) | 205 (175-247) | 206 (175-243) | 0.919 |
| NO.of stent | 1.5±0.8 | 1.6±0.8 | 0.136 |
| Total stent length(mm) | 39.5±24.0 | 41.3±23.4 | 0.196 |
| Mean stent Diameter(mm) | 3.2±1.1 | 3.1±0.5 | 0.001 |

*ACS*, acute coronary syndrome; *CAG*, angiography; *CKD*, chronic kidney disease; *CTO*, chronic total occlusion; *Hb*, hemoglobin; *LMCA*, left main coronary; *LDL-C*, low density lipoprotein cholesterol; *MI*, myocardial infarction; *NSTEMI*, non-ST-segment elevation myocardial infarction; *PCI*, percutaneous coronary intervention; *PLT*, platelet; *UA*, unstable angina pectoris; *WBC*, white blood cell; *STEMI*, ST-segment elevation myocardial infarction
